# *In silico* Strategies to Support Fragment-to-Lead Optimization in Drug Discovery

**DOI:** 10.3389/fchem.2020.00093

**Published:** 2020-02-18

**Authors:** Lauro Ribeiro de Souza Neto, José Teófilo Moreira-Filho, Bruno Junior Neves, Rocío Lucía Beatriz Riveros Maidana, Ana Carolina Ramos Guimarães, Nicholas Furnham, Carolina Horta Andrade, Floriano Paes Silva

**Affiliations:** ^1^LaBECFar – Laboratório de Bioquímica Experimental e Computacional de Fármacos, Instituto Oswaldo Cruz, Fundação Oswaldo Cruz, Rio de Janeiro, Brazil; ^2^LabMol – Laboratory for Molecular Modeling and Drug Design, Faculdade de Farmácia, Universidade Federal de Goiás, Goiânia, Brazil; ^3^Laboratory of Cheminformatics, Centro Universitário de Anápolis – UniEVANGÉLICA, Anápolis, Brazil; ^4^Laboratório de Genômica Funcional e Bioinformática, Instituto Oswaldo Cruz, Fundação Oswaldo Cruz, Rio de Janeiro, Brazil; ^5^Department of Infection Biology, London School of Hygiene and Tropical Medicine, London, United Kingdom

**Keywords:** fragment-based, drug discovery, lead discovery, *in silico* methods, machine learning, *de novo* design, optimization, hot spot analysis

## Abstract

Fragment-based drug (or lead) discovery (FBDD or FBLD) has developed in the last two decades to become a successful key technology in the pharmaceutical industry for early stage drug discovery and development. The FBDD strategy consists of screening low molecular weight compounds against macromolecular targets (usually proteins) of clinical relevance. These small molecular fragments can bind at one or more sites on the target and act as starting points for the development of lead compounds. In developing the fragments attractive features that can translate into compounds with favorable physical, pharmacokinetics and toxicity (ADMET—absorption, distribution, metabolism, excretion, and toxicity) properties can be integrated. Structure-enabled fragment screening campaigns use a combination of screening by a range of biophysical techniques, such as differential scanning fluorimetry, surface plasmon resonance, and thermophoresis, followed by structural characterization of fragment binding using NMR or X-ray crystallography. Structural characterization is also used in subsequent analysis for growing fragments of selected screening hits. The latest iteration of the FBDD workflow employs a high-throughput methodology of massively parallel screening by X-ray crystallography of individually soaked fragments. In this review we will outline the FBDD strategies and explore a variety of *in silico* approaches to support the follow-up fragment-to-lead optimization of either: growing, linking, and merging. These fragment expansion strategies include hot spot analysis, druggability prediction, SAR (structure-activity relationships) by catalog methods, application of machine learning/deep learning models for virtual screening and several *de novo* design methods for proposing synthesizable new compounds. Finally, we will highlight recent case studies in fragment-based drug discovery where *in silico* methods have successfully contributed to the development of lead compounds.

## Introduction

### Fragment-Based Drug Discovery

Since the inception of fragment-based drug discovery (FBDD) over 20 years ago it has become an established technology used in both industry and academia (Hubbard, 2015). FBDD offers an attractive approach for effectively exploring the chemical space for binding a target protein. In conventional high-throughput screening (HTS) campaigns, large libraries of often complex compounds are screened for activity against a target (Hall et al., 2014). In contrast, FBDD use relatively small libraries of low complexity compounds representing fragments of larger more drug-like compounds. By reducing the complexity of the chemicals screened more of the potential binding sites of a target protein can be explored through the binding promiscuity of the fragments (Thomas et al., 2017). Where fragments do bind, albeit with lower potency than the drug-like molecules of HTS, they offer good starting points to design larger higher affinity binders using knowledge of the protein structure as a template to generate compounds with greater ligand efficiency (improved per atom binding energy to the target). This bottom-up approach means that a greater range of chemical space can be explored, leading quickly to higher affinity lead compounds with greater specificity (Patel et al., 2014).

FBDD projects require relatively lower investments in research and development (R&D) than HTS (Davis and Roughley, 2017). An example is the discovery of vemurafenib (Zelboraf^TM^), the first fragment-derived drug, which moved relatively very quickly (6 years) between the phases of R&D pipeline before reaching Food and Drug Association (FDA) approval (Erlanson et al., 2016). Thus, FBDD provides attractive opportunities for the drug discovery field.

### Output of Structure-Enabled Fragment Screening Campaigns

FBDD workflows are multi-step starting with target selection and protein isolation and followed by an initial screen of the fragment library using biophysical techniques such as nuclear magnetic resonance (NMR), surface plasmon resonance (SPR), thermal-shift assay, microscale thermophoresis (MST), mass spectrometry, and others. For fragments which show evidence of binding, a further step of hit validation and characterization occurs principally using X-ray crystallography (Verdonk and Hartshorn, 2004). Using hit characterization, an iterative cycle of fragment development can occur employing a range of *in silico* and experimental techniques. Advances in this protocol try to compress the process by combining the initial fragment screen with the hit characterization. This has been implemented in a high throughput FBDD platform called XChem located at the United Kingdom's national synchrotron the Diamond Light Source (Cox et al., 2016). It uses the ability to produce and handle a large number of crystals of the target protein to screen the fragment library by soaking each individual crystal with a fragment and then using X-ray crystallography to determine which fragments have bound and where. Though this high throughput technique often provides multiple hits, care needs to be taken in interpreting the significance of the hit. Promiscuous fragments may bind parts of the protein which are not involved in the protein function and therefore are unlikely to yield a successful inhibitor. Additionally, as the fragments are by their very nature weak binders and X-ray crystallography being a sensitive technique, observed binding events might be transient and not easily reproducible. It is therefore important to confirm hits with orthogonal structural (e.g., NMR), biophysical techniques (SPR, MST, etc.) or *in vitro* biological assays.

### Fragment Libraries

A crucial step in FBDD process is in the development and choice of the fragment library used in the screening campaign. Several fragment libraries have been developed that exploit certain properties or chemistries. An example of a fragment library is the Diamond-SGC Poised Library (DSPL) (Cox et al., 2016). This has been developed for use with high-throughput XChem platform and consists of around 760 fragments that have been selected to contain at least one functional group that is open to rapid, cheap follow-up synthesis using robust well-characterized reactions (poised) and maximizing chemical diversity. Other fragment libraries optimize other properties such as solubility, 3D traits or based on subsets of existing drugs and related molecules such as natural products (Schuffenhauer et al., 2005). The fragment libraries generally share similar properties of “Rule of 3” compliant i.e., less than 300 Da molecular weight, 3 or less hydrogen bond donors, 3 or less hydrogen bond acceptors and CLogP no more than 3. In addition, they are soluble in dimethyl sulfoxide (DMSO) or phosphate buffered saline. Fragment libraries generally tend to be <1,000 fragments, which is significantly less than the many millions of compounds screened in high-throughput and high content screening campaigns (Trevizani et al., 2017).

### Fragment Expansion Strategies

**Figure 1:**
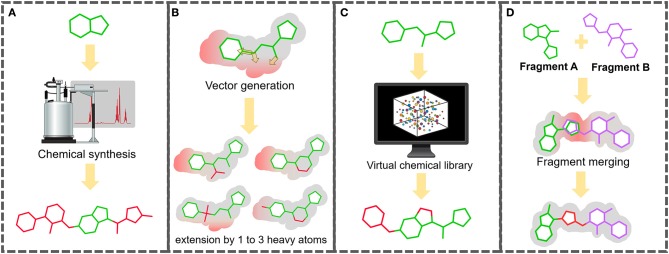
Multiple routes to expanding fragment to more drug-like molecule with improved binding affinity. **(A)** Traditional medicinal chemistry route: knowledge-based design and synthesis. **(B)** “Small steps” route: successive cycles of extension of the fragment hit by 1–3 heavy atoms through vectors defined by high-resolution structural characterization methods, such as X-ray crystallography. **(C)** “Large leaps” or “SAR by catalog” route: from fragment to rule of 5 compliant molecules using virtual screening of commercial compound libraries. **(D)** Fragment merging route: bridging two overlapping fragments bound at neighbor sites. Regardless of the route, expanded fragments should be checked for biological activity using *in vitro, ex vivo*, or *in vivo* assays.

Once the fragment screen has been completed and hits characterized, the next step is the challenge of expanding these fragments to generate larger molecular entities with high binding affinity and demonstrating inhibition activity. There are several strategies that can be followed (Lamoree and Hubbard, 2017) (Figure 1). One option is to use expert medicinal chemistry advice to design and synthesize larger molecules based on the protein and the fragment pose. Another approach is to define vectors along the fragment molecule based on the steric hindrance of the protein target in which the fragment can be expanded. The fragment is then searched for within a large library of synthesizable (or purchasable) molecules which are bigger by between one and three heavy atoms along the identified vectors. These expanded fragments can be synthesized and soaked/co-crystallized and re-screened by X-ray crystallography. Expanded fragments that show improved binding can be further extended or structurally modified using the same process, with this cycle continuing until a larger high-affinity binding entity is reached.

An alternative to this “small steps” approach is to try to get a larger higher affinity binding molecule in a single step. This can be achieved by having an *in silico* method using the fragment in a substructure search of a large purchasable compound library (e.g., Zinc15), and to virtually screen the results using the pose of the fragment to dock the molecules and rank them based on docking parameters (Trevizani et al., 2017). The top-ranking virtual screening hits can then be co-crystallized and well as evaluated *in vitro* and *in vivo*. A final expansion option is to link or merge fragments that hit near to each other or within the same site (Davis and Roughley, 2017). The combined fragments can then be further expanded using the approaches described previously. It is vital that as expansion progresses *in vitro* and *in vivo* assays are conducted to asses activity of the new molecules.

In the next section, we will discuss in depth the main optimization approaches used for a fragment structurally characterized in a binding site of its target. Further sections will describe existing software tools or modeling techniques (e.g., machine learning) employed for taking a fragment hit thorough the path for becoming a lead compound—a process known as fragment-to-lead (F2L)—for drug development and conclude by presenting case studies where *in silico* strategies have been successfully utilized to support the F2L optimization process.

## Fragment Optimization Approaches

**Figure 2:**
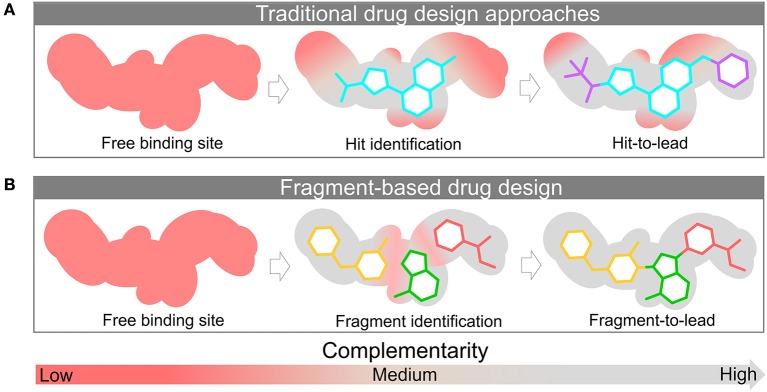
Discovery and structural-optimization of drug-like molecules **(A)** and fragments **(B)** using protein target information. The surface represents the binding site. The red and gray colors represent the level of complementarity of ligand with the active site. Pockets with low complementarity with ligand are colored in red; pockets with high complementarity with ligand are highlighted in gray.

After the hit identification in a FBDD campaign, the fragment moves forward to the optimization phase. This optimization takes into account the structural characteristics of the ligand as well as its binding site. The principle in using fragments relies on the premise that these molecular entities are more efficient ligands compared to drug-like molecules, and their structures can be further optimized more efficiently. In fact, this constitutes one of its many advantages. As small entities, molecular fragments can be iteratively optimized to show a better pharmacokinetic profile in the later development stages. Drug-like molecules may contain functional groups that contribute poorly to protein binding or, in some cases, can even disrupt the protein-ligand interaction. On the other hand, fragments often form high-quality interactions able to more easily bind to the protein target, translating to a greater number of hits. Figure 2 depicts schematically this concept.

Another advantage of FBDD is the potential for faster hit progression through the campaign, since the fragments are usually structurally simple and many follow-up compounds can be easily purchased from commercial databases (e.g., MolPort, ZINC15, and ChemBridge) instead of being synthesized. Another important characteristic often used to defend this approach is the high hit rates. In this sense, high hit rates means that the FBDD yields relatively more hits in comparison to the traditional methods such as HTS (Coutard et al., 2014; Mondal et al., 2015). This is due the inversely related nature between molecular complexity and the binding probability (Hann et al., 2001). Other advantages includes the more efficient chemical space sampling (Coutard et al., 2014; Mondal et al., 2015) and the relative low cost to implement the FBDD, as it can be seen from comparing the usual size of the HTS library (thousands of compounds) with fragment libraries (hundreds of compounds) (Macarron et al., 2011).

Assessment of the interactions between the fragment and its binding site should be carefully performed for further identification of synthetically accessible vectors on the ligand. Although x-ray crystallography data is a valuable technique in fragment optimization, is important to keep in mind that the observed structural data only represents a snapshot of the system under investigation. It's been known that the ligand affinity can be affected by the structural protein dynamics without changing the ligand-binding interface (Matias et al., 2000; Seo et al., 2014). This complex dynamic environment (Henzler-Wildman and Kern, 2007; Boehr et al., 2009) can affect small and weak ligands as fragments. With this in mind, many methods can additionally be used to guide the fragment identification/optimization either providing complementary data (e.g., thermodynamic data) or acting as orthogonal approaches (Ciulli, 2013a). These methods are mostly biophysical (Shuker et al., 1996; Lo et al., 2004; Navratilova and Hopkins, 2010; Pedro and Quinn, 2016) and their use has some advantages such as, direct measurement of the binding, detection of small ligands with low affinity, and not needing any prior information about the protein function (Ciulli, 2013b). Despite the supremacy of biophysical methods, biochemical approaches are increasingly being used (Godemann et al., 2009; Boettcher et al., 2010; Mondal et al., 2015) in FBLD.

In addition to orthogonal and complementary methods, the ligand efficiency (LE) or one of its related metrics should ideally be used to keep track of the quality of follow-up ligands as they progress through the iterative optimization cycle. Some of these parameters are described below.

- **L**igand **E**fficiency (**LE**) (Hopkins et al., 2004; Nissink, 2009; Davis and Roughley, 2017) = ΔG/HAC ^A^;
- **B**inding **E**fficiency **I**ndex (**BEI**) (Abad-Zapatero, 2013) = pKi/MW ^B^;
- **P**ercentage **E**fficiency **I**ndex (**PEI**) (Abad-Zapatero, 2017; Davis and Roughley, 2017) = % inhibition/MW ^B^;
- **S**urface-binding **E**fficiency **I**ndex (**SEI**) (Abad-Zapatero, 2013) = pIC_50_/(TPSA ^C^);
- **Lip**ophilic **E**fficiency (**LipE/LLE**) (Shultz, 2013) = pIC_50_–cLogP;
- **S**ize-**I**ndependent **L**igand **E**fficiency (**SILE**) (Nissink, 2009) = ΔG/HAC^1−*x*^;
- **L**igand **E**fficiency-Dependent **L**i**p**ophlicity (**LELP**) (Davis and Roughley, 2017) = logP/LE.

^A^Heavy Atom Count; ^B^Molecular Weight; ^C^Topological Polar Surface Area;

**Figure 3:**
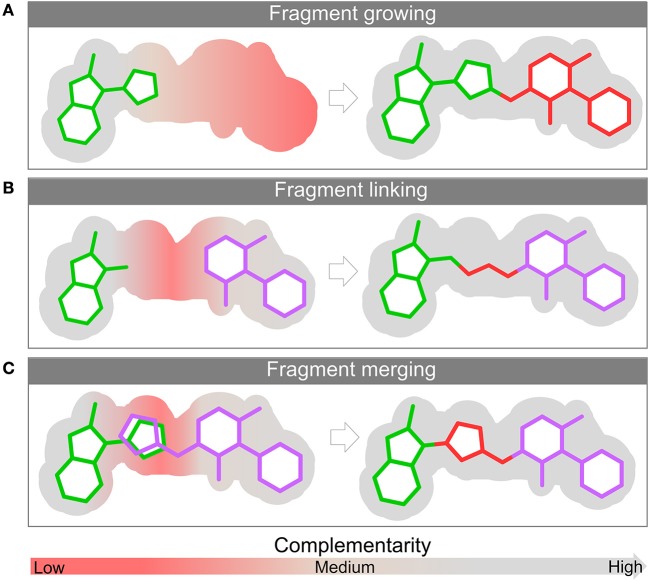
Fragment optimization approaches: fragment growing **(A)**, fragment linking **(B)**, and fragment merging **(C)**. The surface of the binding site is depicted in gray. The red and gray colors represent the level of complementarity of ligand with the active site. Pockets with low complementarity with ligand are colored in red; pockets with high complementarity with ligand are highlighted in gray.

For the sake of brevity these metrics will not be further discussed and we recommend the references above for a deeper understanding. The structural complexity of the protein makes larger, more complex and less efficient molecules less likely to bind. This is one of the main reasons why fragment libraries often yield more hits when compared to a drug-like molecule commonly used in HTS (Hann et al., 2001). The use of fragments is a bottom-up approach, starting from less complex molecules with greater binding efficiency and ending up with a larger optimized molecule. As already highlighted, there are three main strategies that can be employed to optimize a ligand found bound in its target surface: linking, merging and growing (Figure 3). The next sections are dedicated to discussing in more depth each of them.

### Growing

Fragment growing (Figure 3A) is the strategy most commonly employed during FBLD campaigns. As the name suggests, it consists of modifying the fragments to increase their size. Conceptually this approach is identical to the traditional compound modification methods employed in the optimization of hits from HTS campaigns. This modification occurs through the addition of groups.

A recent paper published by Strecker et al. (2019) is an example of how the growing strategy can be used to improve bind affinity. Using computer-aided drug design (CADD) and synthesis, the authors explored small structural modifications in a previously (PDB: 3U0X) identified compound (**1**) (K_i_ = 800 μM).

**Figure 4:**
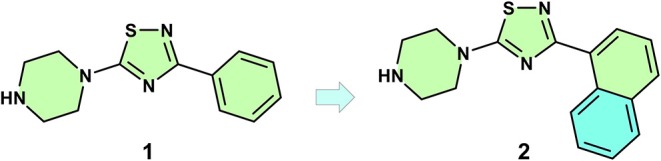
Hit to lead progression of an initial fragment **(1)** to a compound **(2)** with improved affinity.

These studies showed that a modification of a fragment phenyl moiety to a naphthyl allowed two new simultaneous π-π interactions, a parallel-displaced with Trp300 and an edge-to-face with His233. This minor modification led to a compound (**2**) with a 3-fold improved binding affinity (K_i_ = 271 μM) (Figure 4).

This example highlights the use of optimal growth vectors to introduce a rigid group, which led to an increased binding affinity. Alternatively, introduction of a moiety with increased number of rotatable bonds could impact negatively—due to the entropic penalty—in the affinity. Although this optimization approach can be computationally aided without further structural data, small modifications—as in the case of the hypothetical flexible moiety addition—can led to great changes in binding mode. When growing fragments is the chosen approach, structural data can be decisive to avoid misinterpretation.

### Linking

Fragment linking (Figure 3B) describes the process of joining two non-competitive fragments (i.e., fragments that bind in two different sub-pockets of the binding site) with a chemical linker or spacer. Although conceptually simple, linking fragments is perhaps the most challenging strategy to implement. Although fragment linking is the most attractive approach in terms of rapid improvement of potency, the design of a linker with suitable flexibility while not disturbing the original binding modes of the fragments, makes it one of the most challenging optimization approaches.

As previously discussed, the introduction of flexible moieties affects these compounds properties and an optimal orientation should always be pursued. In fact, varying the degree of rigidity of a linker for the purpose of conformational restriction of the linked product can be used as a strategy for linker optimization, as it can be seen in Chung and colleagues work (Chung et al., 2009). This work shows how a conversion of oxime linkers into monoamine and diamines interferes with the rigidity and its impact on binding.

Although often neglected, the impact on the ADMET properties should also be taken in consideration. In the case of the linker, that usually adds rotatable bonds to the system (Ichihara et al., 2011; De Fusco et al., 2017), this modification can lead to poor PK features, like low permeability (Veber et al., 2002).

### Merging

This strategy (Figure 3C) can be used in cases where two distinct fragments partially occupy the same region, or when two binding sites have regions in common and therefore their ligands are partially competitive with respect to the site. In such cases the overlapping parts form a nucleus where dissimilar parts come together. In a recent example, a gain of 2 orders of magnitude in potency was achieved for an inhibitor of flavin-dependent monooxygenase (EthA) transcriptional repressor (EthR) (Nikiforov et al., 2016) where the existence of overlapping groups within fragments bound to EthR allowed the use of merging as an optimization strategy.

Although not always possible, merging is a simpler strategy than linking, as there is no need to design a spacer that joins fragments together (Xu et al., 2017; Miyake et al., 2019). As also seen in this example, like linking (Davis and Roughley, 2017), this approach has the drawback of relying on high-quality structural data to go further in the optimization process.

Therefore, merging is an approach related to the “molecular hybridization” strategy, a long-consolidated approach in medicinal chemistry for designing new compounds with improved potency through the fusion of other active compound structures.

## *In Silico* Strategies for F2L Optimization

### Hot Spots Analysis and Pocket Druggability Prediction

Hot spots analysis is an important tool for structure-based F2L that allows the prediction of the small regions of the binding sites containing residues mostly contributing to the binding free energy (Cukuroglu et al., 2014). Once a fragment hit is experimentally identified, the hot spots analysis can be used to map the subsites around the fragment hit using small organic probes, driving the optimization into higher-affinity ligands (Hall et al., 2012).

One of the most used methods of hot spot analysis is the FTMap web server (Kozakov et al., 2015). This algorithm places 16 small organic probe molecules of different shape, size, and polarity on the protein surface to find favorable positions for each probe. Then, each probe type is clustered and overlapping clusters of different probes, called consensus sites (CSs), represent the hot spots. The consensus sites are ranked by the number of probe clusters, and the main hot spot is, generally, where the fragment hit binds and secondary hot spots are used to extend the fragment in the best direction (Hall et al., 2012; Ngan et al., 2012; Kozakov et al., 2015).

**Figure 5:**
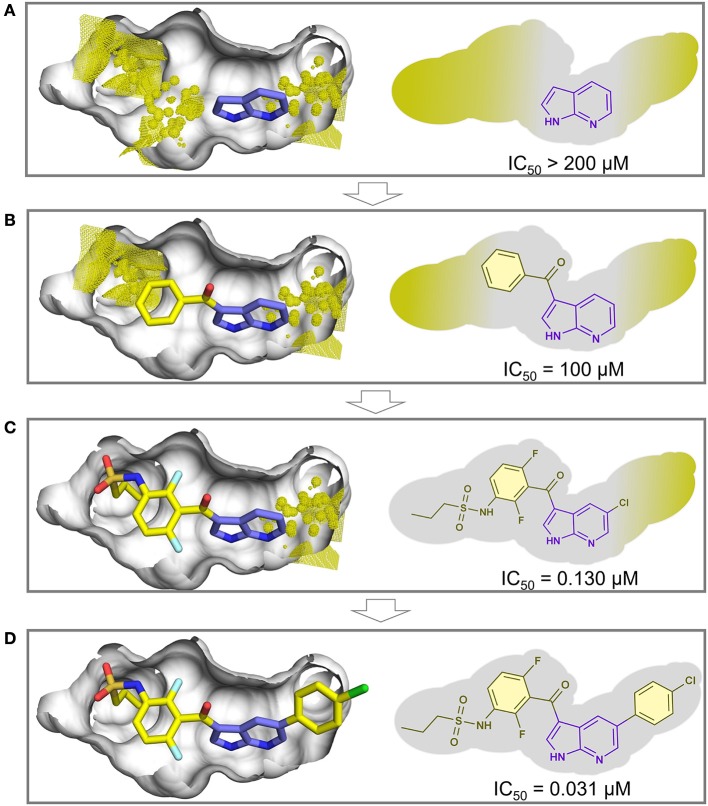
Example of a hot spot analysis using FTMap web server of the oncogenic B-RAF kinase, the target of the first marketed drug from fragment-based drug design, vemurafenib. The surface of the binding site is depicted in gray. **(A)** (PDB ID: 2UVX) the fragment hit (carbon atoms in purple sticks) and the predicted hot spots (yellow dots and surface). **(B–D)** The iterative growing process of vemurafenib (PDB ID: 3OG7) overlapping the predicted hot spots (the carbon atoms of the fragment hit portion is shown in purple sticks and carbon atoms of the grown portions in yellow sticks).

As an example, we used the FTMap server for predicting the hot spots for the oncogenic B-RAF kinase, the target of the first marketed drug from fragment-based drug design, vemurafenib (Bollag et al., 2012). Figure 5A shows the fragment hit experimentally bound to B-RAF kinase (PDB ID: 2UVX) (Donald et al., 2007), and the predicted hot spots around this fragment (shown in yellow dots) using the FTMap server. In Figures 5B–D, the iterative process of growing the fragment hit led to the discovery of the drug vemurafenib (PDB ID: 3OG7) (Bollag et al., 2010) with the hot spots shown in yellow dots. Although hot spot analysis was not used in the F2L process of vemurafenib, the results here showed that the predicted hot spots overlap the grown portions of vemurafenib.

During fragment screening, the fragment hits can bind in different sites of the protein (Giordanetto et al., 2019). If the binding site is not well-defined, the researchers can use the pocket druggability prediction to move forward in F2L with the most druggable site, capable to accommodate ligands orally bioavailable (Schmidtke and Barril, 2010; Hussein et al., 2015). There are many available methods for predicting pocket druggability and these are well-described and reviewed elsewhere (Barril, 2013; Abi Hussein et al., 2017).

### SAR by Catalog

One fast and cheap way in F2L optimization is the SAR by catalog approach (Hall et al., 2017). This approach relies on the search of analogs of *in-house* or commercial databases that can be purchased or rapidly accessed for testing (Schulz et al., 2011). This process can use the fragment hit features for similarity, ligand-based pharmacophores, shape-based, fingerprints (Rogers and Hahn, 2010; Riniker and Landrum, 2013; Alvarsson et al., 2014), and substructure searches to find suitable compounds (Hubbard and Murray, 2011; Andrade et al., 2018). Some databases often used for SAR by catalog are ZINC (Sterling and Irwin, 2015), MolPort (https://www.molport.com), Mcule (https://mcule.com/), and eMolecules (https://www.emolecules.com) that contains collections of commercially available compounds. The databases Enamine (https://enaminestore.com), ChemDiv (http://www.chemdiv.com/) and ChemBridge (https://www.chembridge.com) are direct suppliers.

SAR by catalog approach only retrieves similar compounds or superstructures of the fragment hit. Thus, other filters should be applied to filter compounds with more optimized properties. These filters are molecular docking, ADMET, machine learning models, aqueous solubility, among others, and will be discussed later in this review.

### Molecular Docking

Molecular docking is a computational approach used to predict the position, orientation, and the binding scores of small molecules to proteins (Torres et al., 2019). Hence, as the F2L process is commonly addressed as a combinatorial problem, molecular docking is a method that can be used in combination with other approaches to enhance the F2L process, and to increase the chances to convert a fragment hit into higher affinity ligands. The SAR by catalog approach in combination with molecular docking, for example, can be used to select compounds that maintain the fragment hit binding mode while the binding energy is optimized. Moreover, the number of generated optimized fragments can exceed the number that can be tested experimentally. Thus, applying molecular docking, large compound datasets are efficiently assessed using SAR by catalog, and a small subset of most promising compounds can be selected by binding modes and scores for experimental testing (Grove et al., 2016).

To overcome the problem that SAR by catalog has the limitation to cover only the finite chemical space of commercially available compounds (Hoffer et al., 2018), it is possible to generate virtual catalogs with analogs to hit fragments that can be easily synthesized, astronomically increasing the number of possible compounds. Then, a docking-based virtual screening can be applied to prioritize compounds for experimental evaluation (Rodríguez et al., 2016; Männel et al., 2017).

Another scenario in F2L is when the co-crystallization of a fragment hit commonly fails and no structural information about the binding mode is available. In these cases, alternative strategies for F2L process are required where the binding mode of a fragment can be predicted using molecular docking calculations (Kumar et al., 2012; Chevillard et al., 2018; Erlanson et al., 2019) on high-quality three-dimensional structures of the target in apo form or bound to other ligands. When neither of the latter are available, a theoretical model of the target protein can be obtained by homology modeling methods.

However, there are concerns about fragment docking in the scientific community. The assumption is that fragments, as low molecular weight compounds, are weak binders and promiscuous in binding modes, and consequently, the fragment docking implies in incorrect predictions of the binding modes. Also, there is a concern that scoring functions of the docking programs are parameterized to drug-like ligands, being inaccurate to differentiate native and other low-energy poses (Chen and Shoichet, 2009; Wang et al., 2015; Grove et al., 2016). To overcome these concerns, there are studies demonstrating no significant difference in docking performance between fragments and drug-like ligands (Verdonk et al., 2011; Joseph-mccarthy et al., 2013). They showed that molecular weight is not the principal parameter for docking performance, instead, for high LE compounds the docking performance fared better for both fragments and drug-like ligands (Verdonk et al., 2011; Kumar et al., 2012).

When available, the use of experimental structural information data can be used to support and improve docking performance. These data are used in docking programs including distance constraints, pharmacophore constraints, shape-based constraints, similarity or substructure overlap, interaction fingerprints, hydrogen-bond constraints, and others (Verdonk et al., 2011; Erlanson et al., 2019; Jacquemard et al., 2019).

Similarly to hot spot analysis, molecular docking can also be used to discover secondary binding pockets and guide the F2L process (Männel et al., 2017).

### Machine Learning (ML) and Deep Learning (DL) Models

A large variety of F2L approaches use structure-based methods to optimize fragments into high-affinity ligands taking into consideration the steric and electronic constraints within binding pockets of the target of interest (Schneider and Fechner, 2005). However, the optimized compounds generated constantly present drawbacks of poor synthetic feasibility and/or undesirable biological properties, including absorption, distribution, metabolism, excretion, and toxicity (ADMET) properties (Yang et al., 2019b). In the last years, novel ligand-based methods, including machine learning (ML) models, have been used for F2L campaigns. ML models are statistical methods that present the capacity to learn from data without the explicit programming for this task, and then, make a prediction for new compounds (Mak and Pichika, 2019). The increase of storage capacity and the size of the datasets available, coupled to advances in computer hardware such as graphical processing units (GPUs) (Gawehn et al., 2018), provided means to move theoretical studies in ML to practical applications in drug discovery (Vamathevan et al., 2019).

**Figure 6:**
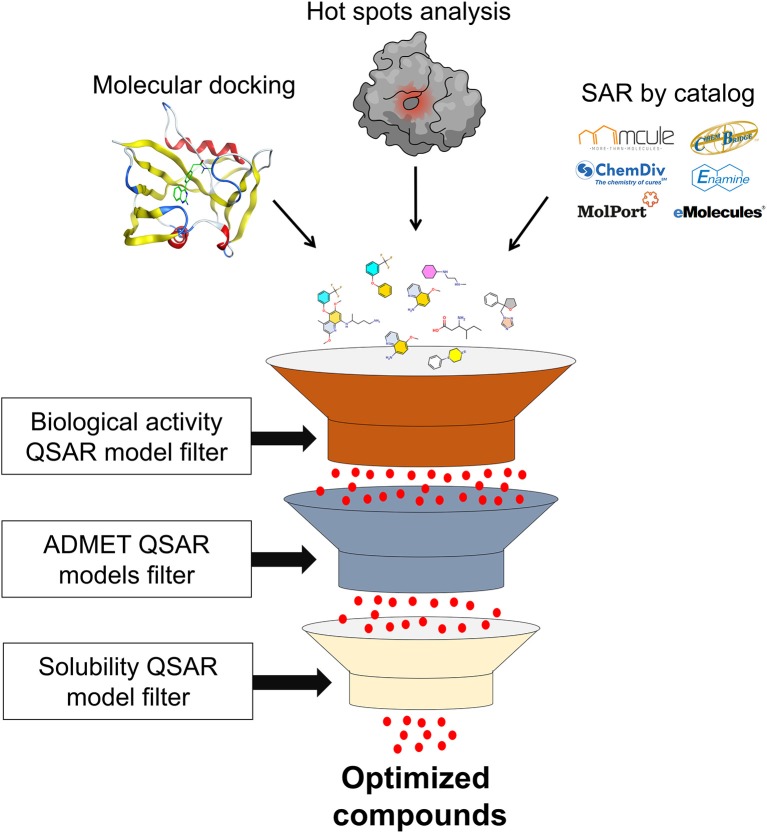
Cascade virtual screening filtering optimized compounds with the desired activities and properties.

The ML algorithms are widely used to construct quantitative structure-activity relationship (QSAR) models, able to find mathematical correlations between molecular features and compound activity/property, and this correlation can be categorical (active, inactive, toxic, nontoxic, etc.) or continuous (pIC_50_, pEC_50_, Ki, and others) by means of classification or regression techniques (Tropsha, 2010; Cherkasov et al., 2014). Thus, machine learning-based QSAR models can be constructed for biological activity, ADMET properties, solubility and synthetic feasibility, among other endpoints, and applied after fragment optimization, with aforementioned methods, in a cascade virtual screening for filtering compounds with the desired activities and properties (Figure 6) (Braga et al., 2014; Neves et al., 2018; Pérez-Sianes et al., 2018).

More recently, a subfield of ML called deep learning (DL) which utilizes artificial neural networks to learn from a large amount of data have been used to resolve complex problems (Mak and Pichika, 2019). DL models are not only able to learn from a dataset and to make predictions for new data but are also able to generate new data instances through a constructive process (Schneider, 2018). In this context, there has been a rising interest in using DL generative and predictive models for F2L optimization (Olivecrona et al., 2017; Gupta et al., 2018).

**Figure 7:**
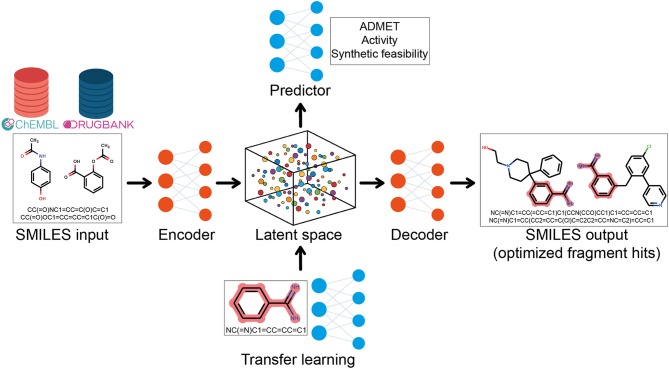
Representation of the integrative approach of generative and predictive deep learning models and transfer learning for fragment-to-lead optimization.

For this task, a combination of DL architectures is used and in many cases, generative DL models based on recurrent neural networks (RNNs) are trained on the simplified molecular input line entry system (SMILES) representation of compounds from large databases (DrugBank, ChEMBL, etc.) to learn the syntax of SMILES language and the chemical space distribution (Olivecrona et al., 2017). After training, the models are able to generate new strings that are new SMILES, corresponding to new compounds (Segler et al., 2018). Then, the transfer learning (TL) can be used to fine-tuning the model and generate compounds related to a fragment hit. As the name suggests, TL learns and transfers the information from an old source to a new application (Yang et al., 2019b). The aim of this integrative approach is to learn general features from a big dataset and, then, retrain the model focusing on a smaller dataset such as fragment hits, for F2L purposes (Figure 7) (Gupta et al., 2018; Segler et al., 2018). Gómez–Bombarelli et al. used variational autoencoder (VAE) to encode SMILES into a continuous latent-space, then a separate multilayer perceptron trained to predict several properties on the latent space was applied to generate new molecules with the desired properties. After this, a decoder was used to retrieve the molecules on the latent space into SMILES (Gómez-Bombarelli et al., 2018). Handling these DL methods in a multidimensional way, fragment hits can be optimized automatically taking into consideration several parameters such as bioactivity, solubility, synthetic feasibility, and ADMET properties, generating new compounds with optimized values for these parameters (Figure 7) (Olivecrona et al., 2017; Ramsundar et al., 2017; Gómez-Bombarelli et al., 2018; Harel and Radinsky, 2018; Li et al., 2018; Merk et al., 2018; Polykovskiy et al., 2018; Popova et al., 2018; Putin et al., 2018; Awale et al., 2019; Vamathevan et al., 2019).

### *De novo* Design

The *de novo* approach looks for new chemical entities from scratch within a structurally defined binding site (Schneider and Clark, 2019). These entities are generated out of building blocks, either by growing from an initial fragment or by linking two or more non-overlapping fragments (Dey and Caflisch, 2008; Kumar et al., 2012). Since their arise, *in silico* methods have played an important role in FBDD (Kumar et al., 2012).

#### Software for Building New Compounds Within a Structurally-Defined Binding Site

*De novo* design software takes advantage of a known binding mode of a fragment, described experimentally or computationally, to propose modified analogs with improved binding affinities. The LUDI (Bohm, 1992) program was one of the first programs developed for *de novo* design. It calculates the interaction sites, maps the molecular fragments, and connects them using bridges, using an empirical scoring. Considering the vast chemical space, evolutionary algorithms are widely used (Srinivas Reddy et al., 2013). In this context, the program GANDI (Dey and Caflisch, 2008) connects pre-docked fragments with linker fragments using a genetic algorithm and a tabu search. The scoring function is a linear combination of force-field binding energy and similarity measures. BREED (Pierce et al., 2004) is a computational method for merging fragments that is widely used. It aligns the 3D coordinates of two ligands and recombines the fragments or substructures into the overlapping bonds to generate new hybrid molecules in a strategy called fragment shuffling. LigBuilder (Wang et al., 2000; Yuan et al., 2011) is a program that uses a genetic algorithm to build up the ligands using a library of organic fragments. It contemplates the growing and linking approach. The 2.0 version includes the synthesis accessibility analysis through a chemical reaction database and retro-synthetic analysis. Autogrow (Durrant et al., 2009, 2013) is another growing approach algorithm that builds a fragment upon a “core” scaffold. The fragment is docked to the receptor. A genetic algorithm evaluates the docking score to select the best population which forms the subsequent generation. The last version considers the synthetic accessibility and the druggability. The program ADAPT (Pegg et al., 2001; Srinivas Reddy et al., 2013) applies a genetic algorithm which uses molecular interactions and docking calculations as a fitness function to reduce the search space. The initial sets of compounds are iteratively built until it reaches the predefined target value.

#### Prediction of ADMET Properties of New Compounds

The ADMET properties and synthetic accessibility (SA) constitutes the secondary constraints whereas primary constraints are geometric and chemical constraints derived from the receptor or target ligand(s) and internal constraints to the geometry and chemistry of the lead compound being constructed. Issues with these points result in the majority of clinical trial failures (Dong et al., 2018). Numerous software and web platforms were developed to predicted ADMET parameters but presented limitations due to narrow chemical space coverage or expensive prices (Cheng et al., 2012). Recent works predominantly rely on ML methods, like random forest (RF), support vector machine (SVM), and tree-based methods (Ferreira and Andricopulo, 2019). The vNN Web Server for ADMET predictions (Schyman et al., 2017) is a publicly available online platform to predict ADMET properties and to build new models based on the k-nearest neighbor (k-NN), which rest on the premise that compounds with similar structures have similar activities. vNN uses all nearest neighbors that are structurally similar to define the model's applicability domain. The similarity distance employed is Tanimoto's coefficient. The platform allows running pre-build ADMET models, and to build and run customized models. Those models assess cytotoxicity, mutagenicity, cardiotoxicity, drug-drug interactions, microsomal stability, and likelihood of causing drug-induced liver injury. Like all machine learning methods, the lack of training data is a limitation.

Pred-hERG (Braga et al., 2015; Alves et al., 2018) is a web app that allows users to predict blockers and non-blockers of the hERG channels, and important drug anti-target associated with lethal cardiac arrhythmia (Mitcheson et al., 2000). The current version of the app (v. 4.2) was developed using ChEMBL (Willighagen et al., 2013) version 23, containing 8,134 compounds with hERG blockage data after curation, using robust and predictive machine learning models based on RF. This app is publicly available at http://labmol.com.br/predherg/.

In admetSAR 2.0 (Cheng et al., 2012; Yang et al., 2019a) tool, the predictive models are built using RF, SVM and kNN algorithms. It presents 27 endpoints and also includes eco-toxicity models and an optimization module called ADMETopt that optimize the query molecule by scaffold hopping based on ADMET properties. The ADMETlab platform (Dong et al., 2018) performs its evaluations based on a database of collected entries and assess drug-likeness evaluation, ADMET prediction, systematic evaluation and database/similarity searching. It uses 31 endpoints applying RF, SVM, recursive partitioning regression (RP), naive Bayes (NB), and decision tree (DT).

SwissADME tool (Daina et al., 2017) uses predictive models for physicochemical properties, lipophilicity and water solubility. It also analyses pharmacokinetics models as BBB permeability, gastrointestinal absorption, P-gp binding, skin permeation (logKp), and CYP450 inhibition. Additionally, the tool presents five drug-likeness models (Lipinsky, Ghose, Veber, Egan, and Muegge) and medicinal chemistry alerts. It is integrated with the SwissDrugDesign workspace. The QikProp (Schrödinger, LLC, NY, 2019) provides rapid predictions of ADME properties for molecules with novel scaffolds as for analogs of well-known drugs and display information about octanol/water and water/gas logPs, logS, logBBB, overall CNS activity, Caco-2 and MDCK cell permeabilities, log Kd for human serum albumin binding, and log IC_50_ for HERG K+-channel blockage.

#### Prediction of Synthetic Tractability (Synthesizability) of New Compounds

Even though large numbers of molecules are generated by *de novo* design, many of them are synthetically infeasible (Dey and Caflisch, 2008). To address this problem, methods to calculate the synthetic accessibility (SA) are being developed. SA can be addressed by estimating the complexity of the molecule or making a retrosynthetic approach, where the complete synthetic tree leading to the molecules needs to be processed (Ertl and Schuffenhauer, 2009). SYLVIA (Boda et al., 2007) is one of the programs that estimate the synthetic accessibility of an organic compound. It obtains the SA score by the addition of five variables as the molecular graph complexity, ring complexity, stereochemical complexity, starting material similarity and reaction center substructure, where the first three are structure-based and the other two utilize information from starting material catalogs and reaction databases. Ertl and Schuffenhauer (2009) developed another method that uses historical synthetic knowledge obtained by analyzing information from millions of already synthesized chemicals and also considers molecule complexity. The method is based on a combination of fragment contributions and a complexity penalty. Podolyan et al. (2010) presented two approaches to quickly predict the synthetic accessibility of chemical compounds by utilizing SVMs operating on molecular descriptors. The first approach (RSsvm) identifies compounds that can be synthesized using a specific set of reactions and starting materials and builds the model by training the compounds identified as synthetically or otherwise accessible by retrosynthetic analysis while the second approach (DRSVM) is constructed to generate a more general assessment. More recently, Fukunishi et al. (2014) designed a new method of predicting SA based on commercially available compound databases and molecular descriptors where the SA is estimated from the probability of the existence of substructures of the compound, the number of symmetry atoms, the graph complexity, and the number of the chiral center of the compound.

#### Synthesizability-Aware Methods

Given the difficulty of synthesis of most of the leads produced by *de novo* approaches, some programs added methods to score the SA. Lead+Op (Lin et al., 2018) is an example of these programs that takes an initial fragment, looks for associated reaction rules, virtually generate the reaction products and select the best binding conformation. Them it generates conformers and select one that becomes a reactant for another round. Also, programs mentioned above as LigBuilder and Autogrow include SA analysis on their current versions. In the medicinal chemistry component of **SwissADME**, a SA score is also included.

Different programs use distinct algorithms for *de novo* design compounds in CADD. Table 1 summarizes some programs cited in this section.

**Table 1:** FBDD programs with respective approaches.

**Program**	**Algorithm**	**FBDD Approach**	**SA**
AUTOGROW	Docking + Genetic Algorithm	Growing	YES (Latest version)
LUDI	Empirical scoring	Linking	NO
AUTO T and T	Transplants fragments into the lead	Merging	NO
LeadOp+R	Looks for associated reaction rules	Growing	YES
GANDI	Genetic Algorithm	Linking	NO
LigBuilder 2	Genetic Algorithm	Linking and Growing	YES
ADAPT	Genetic Algorithm	Growing	NO

## Case Studies in the Last Five Years

### Case 1: FBDD in the Development of New Anti-mycobacterium Drugs

A successful application of the FBDD techniques have been applied to early stage drug discovery of new therapeutics against *Mycobacterium sp*. and in particular *M. tuberculosis* (*Mtb*) and *M. abscessus (Mab)* (Thomas et al., 2017). *Mtb*, the causative agent of tuberculosis, has several therapeutic interventions developed to treat the disease. However, through their long-term use and misuse, the efficacy of these drugs is becoming reduced with strains currently circulating that are mono-resistant, multidrug-resistant, extensively drug-resistant and totally drug-resistant. Despite this little drug development activity has been undertaken since the 1960's. However, relatively in response to the growing drug-resistant threat many different approaches are being deployed to developing novel therapeutics, including FBDD. An example of such an effort is against the meta cleavage product hydrolase (HsaD) that is involved in cholesterol catabolism in *Mtb*. Initial screening was conducted on a library of 1,258 fragments using differential scanning fluorimetry, with hits confirmed by ligand-observed NMR spectroscopy and inhibition by enzymatic assay. The three confirmed fragment initial hits were structurally characterized by X-ray crystallography and fragment soaking. A small series of compounds based on these hits were further tested for activity both *in vitro* and *ex vivo* with promising results (Ryan et al., 2017).

Another target of *Mtb* where FBDD has been applied is the pantothenate synthetase (Pts) where a similar sized fragment library of 1,250 rule-of-three compliant fragments was investigated. An initial screen was performed using a thermal shift assay, followed by a secondary screen using 1-D NMR spectroscopy with ultimate hit validation by isothermal titration calorimetry and characterization by X-ray crystallography. Three distinct fragment binding sites were identified (Silvestre et al., 2013). Follow-up expansion of one of the fragment sites using a combination of fragment linking and fragment growing generated a new series of inhibitors. Though fragment linking seemed to be an attractive approach, the limitation in the repertoire of linkers compromised the binding mode. Greater success came from fragment growth using expert knowledge and the protein target as a template (Hung et al., 2009).

Targets in other pathogenic *Mycobacterium* sp. have also been subject to successful FBDD campaigns. Most notably the recent development of inhibitors against tRNA methyltransferase (TrmD) of *M. abscessus* (Mab). This multi-drug resistant pathogen is increasingly problematic in individuals with cystic fibrosis and other chronic lung conditions. A library of 960 fragments was screened biophysically using differential scanning fluorimetry in a similar fashion used for HsaD, with 53 hits taken to validation and structural characterization using X-ray crystallography (no NMR based validation was undertaken). Only 27 fragments could be validated all of which bound within the substrate binding pocket. A strategy of fragment-merging centered around the overlap of a 4-methoxyphenyl ring system with the indole ring system of two fragments that spanned the adenine and ribose binding pockets. This was explored successfully with a new combined compound providing a new aminopyrazole-indole scaffold with both improved affinity (*K*_d_ = 110 μM, LE of 0.36) and prospects for further elaboration relative to the parent fragments. It also exhibited inhibition activity *in vitro* and *ex vivo* with promising *in vivo* activity also against *M. leprae*, the causative agent of leprosy (Whitehouse et al., 2019).

These successful FBDD campaigns against a range of targets in pathogenic *Mycobacterium* have yielded promising leads with indications of efficacy in *ex vivo* and *in vivo* demonstrating both the power and efficacy of the approach. The ability of these leads to work across a range of pathogens is also highly encouraging. However, work still needs to be done to improve these leads to progress them into early clinical evaluations and into clinical use.

### Case 2: Inhibitors of Dengue Virus Enzymes

A 2014 paper (Coutard et al., 2014) describes the use of FBLD in the discovery of inhibitors for an important subunit of dengue virus (DENV) viral replication complex. In this work, 500 fragments were screened against two subunits of the viral replication complex: NS3 helicase (Hel) and the NS5 mRNA methyltransferase (MTase) subunits. DENV Hel, located in the *C*-terminal region of the NS3 subunit of the replication complex, is involved in viral genome replication and RNA capping. The role of DENV NS5 MTase is related with a double methylation (N-7 and 2'-O) during the cap formation process in flavivirus (Dong et al., 2008).

The authors used a combination of Thermal Shift Assay (TSA), X-ray diffraction crystallography (XRD) and enzymatic assays in order to screen compounds against NS3 DENV Hel and NS5 DENV MTase subunits. The TSA was used as the primary screening technique. During the TSA screening, not surprisingly part of the fragments—used at high concentrations and with poorly optimized physicochemical properties—presented solubility problems. This was the reason for the exclusion of ~4.8% of the screened compounds during this phase. This initial screening yielded 68 hits, from those, 7 were found bound to the DENV MTase subunit by XRD.

Using a direct colorimetric ATPase-based assay to identify inhibitors, from those previous 7 crystallographic hits, 5 fragments (Table 2) were classified as hits with their potency varying between 180 μM and 9 mM.

**Table 2:** Inhibition and potency data from the final hits (Coutard et al., 2014).

	**DENV 2'O-Mtase activity inhibition (%)**	**DENV 2'O-MTase activity IC_**50**_ (mM)**
81	4	3.90 ± 0.16
91	11	2.83 ± 0.18
95	85	0.18 ± 0.01
157	9	9.39 ± 0.90
217	11	3.12 ± 0.27

**Figure 8:**
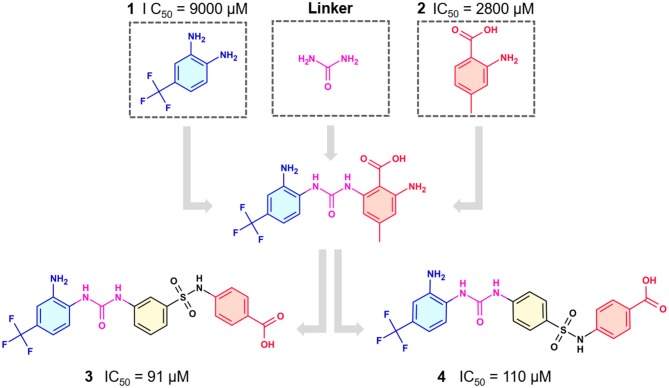
Fragment optimization predecessors and products (Coutard et al., 2014; Benmansour et al., 2017).

In the most recent work, the fragments **3** and **4** (Figure 8) were found bound at the DENV MTase S-Adenosyl-L-methionine (AdoMet) binding site using XRD. A computer-aided fragment optimization gave rise to a new series of compounds using these two fragments. The urea was used as a linker to connect the fragments. Further modifications yielded compounds **5** and **6** (Figure 8).

During the optimization process, the authors had good insights about the important features to the molecule binding on this target. One of these features is the presence of phenyl rings substituted in meta position and is crucial for favoring binding.

This work yielded two inhibitors (**5** and **6**) with potency around 100 μM, even though no effect was observed on a cell assay. Despite this negative result, this work showed the feasibility of the FBDD approach in getting micromolar inhibitors from structurally simple fragments.

### Case 3: MTH1 Inhibitors for Anticancer Drug Discovery

The mutT homolog 1 (MTH1) is an enzyme involved in the prevention of incorporation of deoxynucleoside triphosphates (dNTPs) oxidized by reactive oxygen species (ROS), e.g., 8-oxodGTP or 2-OH-dATP, into DNA, which prevents the killing of the cell. MTH1 is frequently overexpressed in cancer cells and is non-essential in normal cells, proving to be a druggable target for cancer treatment (Smits and Gillespie, 2014; Berglund et al., 2016).

Rudling et al. applied a combination of molecular docking, SAR by catalog, and experimental testing for discovering and optimizing MTH1 inhibitors (Rudling et al., 2017). Initially, a molecular docking-based virtual screening using a crystal structure of MTH1 was performed using 0.3 million fragments from the ZINC fragment-like database, all commercially available. Subsequently, for the 5,000 top-ranked fragments, allowed the search of analogs representing superstructures of the fragment or containing similar substructures in the ZINC database using the chemical structures encoded as circular fingerprints and the Tversky similarity index (Tversky, 1977). The criteria used to select analogs from 4.4 million commercially available compounds in the ZINC database was the following: (i) Tversky similarity >0.8; (ii) up to six additional heavy atoms (HAs) compared to the parent fragment; (iii) improved docking score 80% lower compared to the parent fragment; (iv) visual inspection of the binding modes. After these analyses, a set of 22 commercially available fragments with at least five analogs comprising the above-mentioned criteria were selected for experimental evaluation. Five of these 22 fragments showing IC_50_ values ranging from 5.6 to 79 μM were considered hits and were used for F2L (Table 3). The fragment **7** presented an IC_50_ value of 79 μM and its most potent analog presented an IC_50_ of 170 nM, representing a 470-fold improvement (Table 3). Although the crystal structure of the fragment **7** in complex with MTH1 was not obtained, the crystallization of its most potent analog and MTH1 was solved at 1.85 Å resolution, demonstrating an RMSD of 0.6 Å between the common atoms of the fragment **7** (binding mode predicted by molecular docking) and the analog (crystal structure). Because of the closely related structures and binding modes of fragments **8** and **9**, its analogs were analyzed together, but the most potent analog presented an IC_50_ of only 3.5 μM. The most potent analog of fragment **10** presented a 190-fold increase of the activity, with an IC_50_ of 120 nM (Table 3). The crystallization of fragment **10** with MTH1 was also unsuccessful, but the molecular docking was able to predict the binding mode, showing an RMSD of 0.9 comparing the overlapping atoms with the crystal of the most potent analog obtained at 1.50 Å resolution. The analogs of the fragment **11** were not available during the study. This work demonstrated that virtual screening and SAR by catalog can be used to rapidly identify and optimize fragments into nanomolar inhibitors (Rudling et al., 2017).

**Table 3:** Experimental data for the five most potent MTH1 inhibitors (data taken from Rudling et al., 2017).

**Fragment ID**	**Fragment 2D structure**	**Fragment IC_**50**_**	**Most potent analog 2D structure**	**Analog IC_**50**_**
7	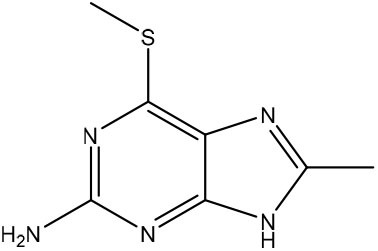	79 μM	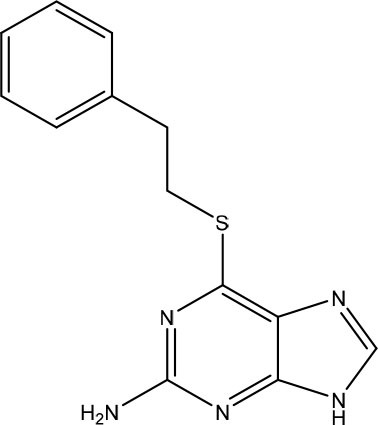	0.17 μM
8	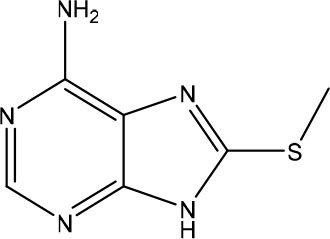	24 μM	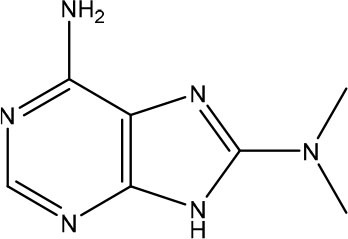	3.5 μM
9	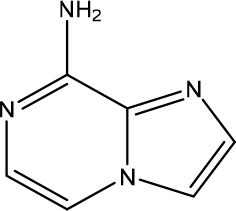	26 μM	n/a	n/a
10	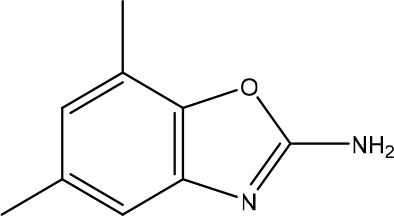	23 μM	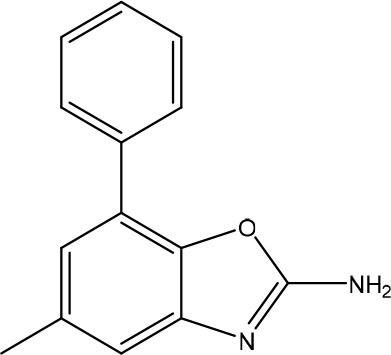	0.12 μM
11	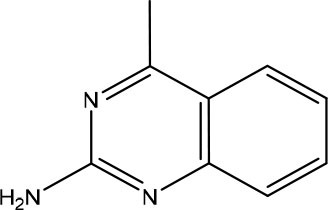	5.6 μM	n/a	n/a

*n/a, not available*.

### Case 4: New Acetylcholinesterase Inhibitors Against Alzheimer's Disease

Alzheimer's disease is a neurodegenerative disorder and has no cure. The actual treatments are based on drugs that leverage the transmission of electrical impulses. Pascoini et al. (2019), computationally developed new acetylcholinesterase (AChE) inhibitors. AChE is responsible for decreasing levels of acetylcholine in the synaptic cleft. Their inhibition enhances the transmission of the electric impulse (Polinsky, 1998; Talesa, 2001).

For the *in silico* inhibitor development, they divided the process into four steps. First, a *de novo* design was applied to generate an initial library of compounds. The first library was then filtered according to ADME properties at the second step. In the third step, the filtered library was filtered again using a similarity criterion. Finally, the resulting library was used in docking studies. The best three complexes were used for molecular dynamic studies. In this work, they used three reference drugs for AD treatment: donepezil, galantamine, and rivastigmine.

For the *de novo* design, the LigBuilder software was used. The CAVITY procedure was employed to detect and analyze ligand-binding sites of the target. It classified the cavities' druggability, who would be used for docking studies. The BUILD procedure was used in the exploring and growing/linking modes. In the explore mode, fragments from the program's database were added in the protein site and their interaction was scored. Then, the fragments with the best scores were linked. In the growing mode, seeds molecules were put at the binding site and fragments were added to the seeds. At the linking mode, the seed was divided into fragments and other fragments were added to them. After the BUILD procedure, they got a library of 2.5 million compounds. The resulting library was filtered according to ADME properties with the software QUIKPROP where molecules that infringed more than five properties (physicochemical properties, lipophilicity, water solubility, pharmacokinetics models) were discarded. A library of 6,000 compounds results from this process. After this, the Tanimoto's coefficient was applied to measure the similarity among the molecules. Molecules below 0.85 were excluded and 1,500 molecules were considered for the next step. The final step consisted of docking studies, carried out with the GLIDE software and the Induced Fit Docking protocol. Afterward, they selected the three best complexes from the docking and used them as input structures for molecular dynamic studies. Finally, they obtained three compounds with high stability and good binding energies, some of them even better than the reference drugs.

## Concluding Remarks

FBDD has matured to become a key strategy in modern pharmaceutical research. With less requirement for large chemical libraries and the possibility of using a range of biophysical methods for screening, the easier and scalable implementation of this strategy has facilitated its popularization, especially among academic institutions and smaller pharmaceutical companies.

The main reason for the success of the FBDD strategy is because it presents a more efficient and consistent route for optimization of initial screening hits into lead compounds. As reviewed here, many routes are available for expansion of fragment hits and *in silico* methods are key to support or guide the majority of them.

A variety of *in silico* methods have been used in F2L optimization in FBDD, from binding site analysis to *de novo* design of new fragment-derived ligands with synthesizability-aware methods. The case studies highlighted here clearly demonstrate how the different *in silico* methods can be used in integrated form and combined with experimental approaches to successfully develop higher affinity ligands from fragments.

Advances in artificial intelligence methods, such as deep learning, hold a great potential to accelerate the optimization of fragment hits in lead compounds. Recent examples show that these hits can be already optimized automatically taking into consideration several parameters such as bioactivity, solubility, synthetic feasibility, and ADMET properties.

## Author Contributions

FS, CA, and NF drafted the review topics with the abstract and revised the manuscript. FS drafted the conclusion and contributed to several parts of the manuscript. NF drafted the introduction and one of the case studies. LS drafted the fragment optimization section and one of the case studies. JM-F drafted most of the *in silico* strategies section and one of the case studies. BN designed all figures and revised the manuscript. AG and RM drafted the *de novo* design section and one of the case studies.

### Conflict of Interest

The authors declare that the research was conducted in the absence of any commercial or financial relationships that could be construed as a potential conflict of interest.
